# Editorial: The role of natural and synthetic antioxidants in the therapeutic targeting of oxidative stress

**DOI:** 10.3389/fmolb.2025.1630095

**Published:** 2025-06-10

**Authors:** Alessia Remigante, Sara Spinelli, Rossana Morabito, Valentina Mittova

**Affiliations:** ^1^ Department of Biomedical, Dental and Morphological and Functional Imaging, University of Messina, Messina, Italy; ^2^ Department of Chemical, Biological, Pharmaceutical and Environmental Sciences, University of Messina, Messina, Italy; ^3^ Scientific‐Research Institute of Experimental and Clinical Medicine, University Geomedi, Tbilisi, Georgia

**Keywords:** ROS, plant-derived antioxidants, synthetic antioxidants, oxidative stress, therapeutic agents

Oxidative stress is involved in a wide range of diseases, including neurological (Pizzino et al., 2017), cardiovascular (Valaitienė and Laučytė-Cibulskienė, 2024), and pulmonary diseases, diabetes and cancer (Forman and Zhang, 2021). However, attempts at the prevention and treatment of such diseases using small-molecule antioxidants have often been unsuccessful. It was then suggested that the focus should be shifted towards disease-specific and target-directed therapy (Firuzi et al., 2011), leading to antioxidant compounds being accepted for clinical use. This Research Topic includes studies exploring the role of natural and synthetic antioxidants in the therapeutic targeting of oxidative stress, assessing the efficacy and the mechanisms by which antioxidants exert their effects.

Plant-derived antioxidants are a large group of natural compounds possessing ROS-scavenging activity (Remigante et al., 2023; Pirtskhalava et al., 2024). Despite the accumulated data on antioxidant structures and properties, the bioactivity of many natural compounds remains unknown. The action mechanism and therapeutic potential of licochalcone B, a plant-derived chalcone, were examined in the extensive review by Shaikh et al. Licochalcone B has antioxidant properties, possessing anti-inflammatory, anti-cancer, hepatoprotective, and neuroprotective effects. These diverse pharmacological activities were linked to autophagy, apoptosis, inflammation, and oxidative stress. The signalling mechanisms that modulated these responses were identified, and the potential of licochalcone B as a lead compound for drug discovery was demonstrated.

The review of Ghosh et al. discusses the biological activities of *Vaccinium*-derived anthocyanins (quercetin, myricetin and their glycosidic derivatives), which demonstrated cardioprotective, antidiabetic, anticancer, neuroprotective, anti-inflammatory, antimicrobial and antiviral effects. The review discusses the experimental results, which indicate that total anthocyanin, phenolic and antioxidant contents may be associated with the berry’s effectiveness. However, challenges regarding *in vitro* and *in vivo* studies for elucidating the mechanism of action and health benefits for various groups of patients remain.

The action of another natural antioxidant, flavonoid chrysin, against the effects of organophosphorus pesticide methidathion on ovaries was demonstrated in the study of Hamed et al. Chrysin efficiently alleviated pesticide-induced ovarian damage, as was demonstrated by significantly improved lipid peroxidation and the content of non-enzymatic and enzymatic antioxidants, preventing hormonal imbalance and histopathological changes in methidathion-treated rats.

For the first time, the antimalarial potential of bioactive constituents derived from the bark of *Schleichera oleosa* was reported in the study of Vanaja et al. The novel compounds scillarenin, possessing antioxidant properties and 4-[(Z)-(6-hydroxy-3-oxo-1-benzofuran-2(3H)-ylidene) methyl] phenyl beta-D-glucopyranoside were identified, using GC-MS and LC-MS. Molecular docking confirmed the strong binding affinities of these compounds to malaria target proteins 1CEQ and 4ZL4, surpassing some standard drugs in efficacy.

Since oxidative damage is strongly implicated in the pathogenesis of neurodegenerative (Kamat et al., 2008) and neuropsychiatric diseases (Pandya et al., 2013), the mechanism of oxidative damage and the role of antioxidants in the prevention and treatment of these diseases were explored in several studies.

Thus, the review by Yao et al. discusses the role of potential oxidative biomarkers in the prognosis of intracerebral haemorrhage (ICH) and the development of antioxidative treatments. The higher levels of oxidative stress indicators (malondialdehyde, hydroxy-2-nonenal, F2 isoprostanes, and ischemia-modified albumin) were detected in ICH patients. ICH also increased the activity of antioxidant enzymes (SOD, CAT, GPX) and non-enzymatic antioxidants (vitamins A, E and C). However, the results of ROS-targeted therapy of ICH did not demonstrate high effectiveness. It was suggested that the successful treatment of ICH requires an increase in the degradation and neutralisation of ROS, which could be achieved via modulation of several signalling pathways (Nrf2/ARE, Wnt/β).

Liu et al. demonstrated the role of oxidative stress in the pathogenesis of schizophrenia, major depressive disorder, anxiety and bipolar disorder and explored the role of antioxidants in the prevention of these diseases. The functions of such antioxidants as N-acetylcysteine, sulforaphane, alpha-lipoic acid, L-carnitine, ascorbic acid and flavones in the alleviation of symptoms of neuropsychiatric disorders via the provision of antidepressant, neurotropic and anti-inflammatory effects were discussed. The authors concluded that antioxidants maintain the normal functioning of the nervous system by regulating the balance of neurotransmitters, inhibiting inflammatory responses and improving the efficiency of the mitochondrial energy metabolism.

Micucci et al. assessed the toxicity of Q10-diacetate, a derivative of coenzyme Q10, on the mouse hippocampal neuronal cell line under rotenone-induced oxidative stress. Q10-diacetate possesses antioxidant properties and is not toxic under normal conditions. Q10-diacetate reduced superoxide production, enhanced the expression of mitochondrial biogenesis genes, and contributed to sustained ATP synthesis under oxidative stress conditions. These findings indicate the need for an *in vivo* investigation into the pharmacokinetics of Q10-diacetate for therapeutic application.

Several studies revealed the association between the reduced expression and activity of antioxidant enzymes and the development of vascular diseases (Radovanovic et al., 2021), but the data on the use of antioxidant enzymes as markers of such disease are contradictory. The clinical study by Chung et al. performed on randomly selected immunosuppressive drug-naïve patients with antineutrophil cytoplasmic antibody (ANCA)-associated vasculitis (AAV) demonstrated for the first time that the activity of the one of first-line antioxidant defence enzyme, serum glutathione peroxidase isoenzyme (GPX-3) correlates with vasculitis activity and was significantly lower in patients with general AAV manifestation in comparison with those without. The study suggests using serum GPX-3 as a complementary biomarker for assessing AAV activity in clinical practice.

The magnetically targeted nanocomposite for the delivery of hydrophobic antioxidant curcumin and a calcitonin gene-related peptide (CGRP) antagonist (CGRPi) for the treatment of neuropathic pain was obtained in the study of Zhu et al. Curcumin provides an analgesic effect, and CGRP expression levels increase after nerve injury. An *in vivo* study demonstrated that the drug-delivery system can effectively enter the site of sciatic nerve injury in mice, alleviating the injury, inflammatory and neuropathic pain response. The introduction of nanocomposites also inhibited oxidative stress and ROS production, indicating the feasibility of the application in the treatment of neuropathic pain.

The studies presented in this Research Topic provide important insights into the structure and mode of action of synthetic and natural antioxidants. We are grateful to all authors, reviewers, and contributors who helped to make this Research Topic a success. Their studies have increased our comprehension of the role of antioxidants as therapeutic agents and enabled future developments in this field.
